# The Effect of a Mindfulness-Based Intervention on Attention, Self-Control, and Aggressiveness in Primary School Pupils

**DOI:** 10.3390/ijerph17072447

**Published:** 2020-04-03

**Authors:** Zara Suárez-García, David Álvarez-García, Patricia García-Redondo, Celestino Rodríguez

**Affiliations:** Faculty of Psychology, University of Oviedo, Plaza de Feijoo, s/n, 33003 Oviedo, Spain; alvarezgardavid@uniovi.es (D.Á.-G.); garciarpatricia@uniovi.es (P.G.-R.); rodriguezcelestino@uniovi.es (C.R.)

**Keywords:** mindfulness, intervention, attention, self-control, aggressiveness, school

## Abstract

The objective of this study was to examine the effect of *Mindkeys Training*, a mindfulness-based educational intervention, on attention, self-control, and aggressiveness in third-year primary school pupils. In order to achieve this aim, a switching replications design was used. Two groups of third year primary students (n_GE1_ = 40; n_GE2_ = 33), aged between 7 and 10 years old (M = 8.08; DT = 0.49), had the intervention at different time points, such that while one served as the experimental group, the other served as the control group. Longitudinal differences were examined in both groups, and cross-sectional differences were examined between the two groups at three time points; at the start of the study, and following the intervention with each group. To that end, measurements of problems of attention, deficits of self-control, and aggressiveness for students were obtained via a teacher rating scale. The intervention program demonstrated a positive effect on the reduction of pupils’ attention problems, deficits of self-control, and aggressiveness. The effects were greater on the cognitive variables that the intervention worked on directly (attention and self-control). Attention was the variable on which the intervention exhibited the longest term effects.

## 1. Introduction

One of the main objectives of primary schools is to maintain a good learning environment, in which the children pay proper attention during tasks and explanations, behave thoughtfully, and interact with each other appropriately. During this educational stage, their capacity for attention and self-control and their social skills are still not fully developed. The gradual development of the brain, along with children’s experiences and learning, encourage the progressive improvement of these skills from infancy to adulthood [1].Childhood, therefore, is a fundamental stage in the development of these skills, and schools are a particularly important context for their development.

Interventions for developing these kinds of cognitive and social skills can be made from various approaches. Among them, in recent years, mindfulness-based interventions have been the subject of growing attention. The concept of mindfulness refers to both a mental state and a set of practices that are characterized by two components: the self-regulation of attention, so that it is maintained on immediate experience, thereby allowing for the increased recognition of mental events in the present moment; and the adoption of an orientation towards one’s experiences in the present moment, characterized by curiosity, openness, and acceptance [2].

Mindfulness-based interventions refer to any treatment that intentionally trains these two components as the core therapeutic element for reducing problem behavior or increasing wellbeing behavior [3]. Although, initially, these kinds of interventions were done with adults in medical settings, they are increasingly being applied to children and young people in school settings. There are significant differences between the various published mindfulness-based interventions aimed at schoolchildren, mainly around who applies the intervention, the types of activities, and how long the intervention lasts.

The two main possibilities for who applies the intervention in school are trained teachers and external experts. Meta-analyses have indicated that the effect of the intervention is more positive at follow-up when trained teachers deliver the program [4]. Teachers remain with their students in the classroom upon completion of the intervention, so that it is likely that they continue incorporating elements of the intervention with their students.

The types of activities include breathing exercises (deep breathing with breath awareness); body scan (awareness of the body, focusing on each part of the body); body practices such as yoga (breathing, body awareness); the conscious practice of routine activities; loving-kindness meditation (a meditation practice to accept, love, and show kindness to oneself and others); and working with thoughts and emotions [5]. These programs are more effective when they combine different types of mindfulness activities [4].

The duration mainly depends on the age of the target group and the objective of the intervention. The ranges of the numbers of sessions and hours of effective mindfulness-based interventions are very large. However, the most common length in school settings is 5–10 weeks [4]. In any case, it is preferable to have short, daily intervention sessions, rather than longer, weekly sessions [6].

Recent meta-analyses indicate that mindfulness-based interventions in the school context have a positive, generally moderate effect on improving students’ skills, and reducing their problem behaviors. Zenner, Herrnleben-Kurz, and Walach concluded that mindfulness-based training in a school context had moderate-to-large effects on cognitive performance (mainly attention), and small but significant effects on stress, coping, and resilience [7]. Carsley, Khoury, and Heath concluded that mindfulness interventions were helpful, with small-to-moderate effects. They detailed that the effects were greater in late adolescence (age 15–19) than middle childhood (age 6–10), and that interventions were more effective when different types of mindfulness activity were combined [4].

Although the effects of mindfulness-based intervention programs are clearer on the cognitive dimensions that are directly worked on (mainly the self-regulation of attention), some meta-analyses have indicated positive effects improving pro-social behavior [8] and reducing disruptive behavior [9]. The meta-analysis conducted by Donald et al., who did not look specifically at children or at the school context, found a greater effect the older the target group—up to adulthood—and concluded that according to the evidence, these effects may occur via greater empathic concern, emotional regulation, and positive affect [8]. The meta-analysis conducted by Klingbeil et al. was focused on single-case studies with children and adolescents, with intervention at home or at school. They recognized that with the data provided by the studies, it was difficult to determine whether the observed changes in behavior were due to increased mindful practice or another component of the intervention (e.g., adult attention) [9].

The meta-analyses we reviewed suggest, therefore, that mindfulness-based interventions in schools have demonstrated positive effects in students, but rigorous studies are scarce, and the efficacy of these programs needs to be the subject of continued study. The objective of this study was to examine the effect of an educational mindfulness-based study on attention, self-control, and aggressiveness in a sample of primary school pupils. It is expected that the mindfulness-based intervention program designed and tested in this study will contribute to improved attention and self-control, along with reduced aggressiveness in the pupils.

## 2. Materials and Methods

### 2.1. Research Design

To test the intervention program, we used a switching replications design [10]. The target students for the intervention were split into two groups. This division was not random, as it followed the natural organization of the class groups. Both groups were evaluated at three time points. Between the first and second time points, only one group received the intervention; in other words, one group was the experimental group and the other the control group. The group that received the intervention first was called Experimental Group 1 (EG1). Between the second and third time points, the roles of experimental and control groups were swapped. The group that received the treatment at that moment was called Experimental Group 2 (EG2) (Table 1).

### 2.2. Participants

The sample of pupils who were the target of the intervention was made up of 73 third year primary school pupils, aged between 7 and 10 years old (M = 8.08; SD = 0.49). Third grade was chosen because it is a moment when attention and self-control abilities are in development, students can understand and follow the proposed explanations and activities, and there can be continuity in the application of these types of task to these children in subsequent school years if desired. The pupils belonged to three schools in Asturias (Spain). All of the three schools were state, urban, and middle socioeconomic status schools. The sample was split into two groups. EG1 was made up of 40 pupils from three classes in two schools. EG2 was made up of 33 pupils from two classes in a single school.

The main teacher for each class also participated—the teachers with the most contact with and best knowledge of the pupils in the study. The five teachers had more than 10 years of teaching experience and had known the pupils since the start of the school year. The EG1 teachers were two women and one man; the EG2 teachers were one woman and one man.

### 2.3. The Intervention Program

The mindfulness-based intervention program to be tested is called *Mindkeys Training*. It was designed for primary school students, using a methodology and terminology adjusted to this educational stage. This intervention program consists of training aimed at teachers and pupils.

The teachers were trained first (Table 2). A mindfulness expert conducted two 1-hour training sessions with the teachers during their working day to explain what mindfulness was and how it could be applied in the classroom. Basic techniques were taught based on breathing awareness and consciously doing routine activities. Lastly, the activities that they would have to do in the classroom before the beginning of the eight-week program aimed at the pupils, which was the core of the intervention, were explained.

During the three weeks following their final training session, the teachers added three types of activities to their classrooms (Table 3). These activities were done every day, with a different activity being done each week.

Following that, the pupils’ mindfulness-based training took place. A mindfulness expert conducted 8 sessions at the school, lasting one hour each, on the first day of each week, during the school day. The 8 sessions had the following generic structure: (1) Sounding a singing bowl (to start the session) and silence; (2) The explanation of a breathing technique; (3) An attention to sound exercise; (4) Reading a story and a debate; (5) A specific activity and an explanation of the week’s challenge; and (6) Sounding the singing bowl (to end the session). Table 4 gives the specific content and activities for each week.

On the other days of each week, the teachers directed reinforcement sessions for the pupils, each lasting 10 min. These sessions were structured as follows: (1) Sounding the singing bowl (to start the session) and silence; (2) A correct posture and attention to sound exercise; (3) Practicing the breathing technique learned that week; (4) Doing the week’s challenge activity; and (5) Sounding the singing bowl (to end the session).

### 2.4. Measuring Instruments

Five measuring instruments were used in this study: three scales for teachers and two tests for students. The three scales for teachers were used to test the effect of the intervention program, regarding the three dependent variables analyzed (attention problems, self-control deficits, and aggressiveness in their pupils). The two tests for children were used to identify possible initial differences between both experimental groups. The intellectual ability test was used as an indicator of learning potential and therefore as an indicator of the ability for getting benefit from the training. The attentional ability test is an objective measure of attention. It complements the information from teachers and allows checking if teachers’ ratings are in accord with the objective execution of the students.

#### 2.4.1. Teachers’ Measures

*Attention problems*. The possible presence of attention problems in pupils was evaluated via five items from the “Attention Problems” subscale of the primary school teachers’ version of the “Evaluation System for Children and Adolescents” (SENA; [13]). We used items 17, 57, 66, 100, and 106 from the original scale (e.g., “It is hard for them to keep their attention on what they are doing”). The items have a five-option Likert-type response (from 1—never or almost never, to 5—always or almost always). The total score in this factor is the sum of the individual item scores (minimum 5, maximum 25). High scores indicate high levels of attention problems. The internal consistency of the scores on this subscale, in the sample in this study, was high (α = 0.90).

*Self-control deficits.* Possible deficits in pupils’ self-control (cognitive and motor) was assessed using five items from the “Hyperactivity-impulsivity” subscale from the primary teachers’ version of SENA [13]. We used items 9, 16, 63, 101, and 128 from the original scale (e.g., “It is hard for them to wait their turn”). The items have five-option Likert-type responses (from 1—never or almost never, to 5—always or almost always). The total score in this factor is the sum of the individual item scores (minimum 5, maximum 25). High scores indicate high levels of self-control deficits. The internal consistency of the scores on this subscale, in the sample in this study, was high (α = 0.84).

*Aggressiveness*. The possible presence of aggressive behaviors in pupils was assessed using five items from the “Aggression” subscale from the primary teachers’ version of SENA [13]. We used items 38, 60, 77, 93, and 125 from the original scale (e.g., “They hit other children”). The items have five-option Likert-type responses (from 1—never or almost never, to 5—always or almost always). The total score in this factor is the sum of the individual item scores (minimum 5, maximum 25). High scores indicate high levels of aggressiveness. The internal consistency of the scores on this subscale, in the sample in this study, was high (α = 0.96).

#### 2.4.2. Child Measures

*Intellectual ability*. We used the *Factor G* test (Scale 2—Form A), from [14], to get a score of the pupils’ general intelligence. This is made up of four tests: series (12 items), classification (14 items), matrices (12 items), and conditions (8 items). Each correct answer is worth one point. The total score ranges from a minimum of 0 points to a maximum of 46. The tasks making up the test are non-verbal and culture-free. The test score offers a measure of the fluid intelligence of the pupil. A high score reflects a good natural aptitude for doing tasks requiring cognitive aptitude—good learning potential. The reliability, obtained via the two-halves method, correlating the score from the even numbered elements with the score from the odd numbered elements, was 0.81 in the validation sample.

*Perceptive and attentional ability.* We used the *Test of Perception of Differences-Revised* (CARAS-R; [15]) to obtain an objective measure for each student in a task requiring the ability for perceptive discrimination and sustained selective attention. The CARAS-R test has 60 elements, each one with three drawings of faces made with basic strokes (hair, eyebrows, eyes, and mouth). In three minutes, the test-taker has to indicate, for each element, which face is different to the other two. One piece of data provided by the CARAS-R is the net number of correct answers (the number of correct answers minus the number of errors). The maximum possible score is 60 and the minimum is −60. High scores indicate good visual-perceptive and attentional abilities. The reliability of the scores in the current study (net correct answers), analyzed by test-retest, was 0.84.

### 2.5. Procedure

Firstly, the intervention program was designed and the measuring instruments were selected. No modifications were made to the original tests for the pupils (Factor G and CARAS-R). For the teachers’ questionnaire, a short 15-item test was created by taking only the items referring to “Attention problems”, “Hyperactivity-impulsivity”, and “Aggression” from the primary school teachers’ version of SENA; and reducing the number of items to 5 per factor, considering their factorial loadings in prior validations of the test and their content, omitting items which were redundant according to three experts.

The research team contacted three state-funded schools in Asturias who had expressed an interest in applying a mindfulness-based intervention. The headteachers of each school were informed of the study objectives and procedures; the voluntary, anonymous nature of the study; and the confidential treatment of the results. The pupils’ parents or guardians were informed of the activity and were asked to provide their consent for their children to participate. This study was part of a larger study about improvement in the school climate, which was approved by the Research Ethics Committee of the Principality of Asturias. The study was performed in accordance with the ethical standards as laid down in the 1964 Declaration of Helsinki and its later amendments.

The study was performed during academic year 2018/2019. In Spain, academic years go from September to June, and are divided into three terms. The students in two of the schools (EG1) received the intervention during the second term (January to March 2019) and the students from the third school (EG2) received it in the third term (March to June 2019). Therefore, in this way, all of the pupils taking part received the programmed intervention. Each school was randomly assigned to an experimental group.

The measuring instruments were applied at three time points (Table 5). *Observation 1* took place in January 2019, at the beginning of the second term; *Observation 2* in March, at the end of the second term; and *Observation 3* in June, at the end of the third term. The student instruments (Factor G and CARAS-R) were only applied in Observation 1. The research team applied them in class, during school hours, to the class group. Before completing the questionnaires, the students were informed that it was anonymous and confidential, and that their participation was voluntary. The teachers’ questionnaire (15 items from three subscales from SENA) was applied at the three time points. Each teacher gave every student a score at each time point. The research team gave the teachers instructions about completing the test. The teachers completed the test during non-teaching time.

### 2.6. Data Analysis

To examine the efficacy of the intervention, we compared the scores of the three adapted SENA subscales (the teachers’ questionnaire) cross-sectionally and longitudinally at the three evaluation time points. The cross-sectional analysis compared the scores for the two groups (EG1 and EG2) at each time point. The longitudinal analysis compared each group’s score with their score at the previous time point. For the variables Attention problems and Self-control deficits we used parametric statistics (Student’s t test for independent samples, and Student’s t test for related samples). For the variable Aggressiveness, the distribution of which was not close to normality, we used non-parametric statistics (the Mann-Whitney U and Wilcoxon tests). To examine the effect sizes of the differences, we used Cohen’s *d*. Normally, the effect size is considered small for values of 0.2, moderate at 0.5, and large at 0.8 [16].

To examine whether the groups differed at the beginning, in addition to the scores in the three SENA subscales provided by the teachers’ evaluations at Observation 1, at the same time point, we also measured students’ intellectual, perceptive, and attentional abilities via the tasks in the Factor G and CARAS-R tests. The Factor G raw score and the net correct response score (correct minus errors) in CARAS-R were compared between EG1 and EG2, statistically controlling for the possible effect of age, in both cases using analysis of covariance (ANCOVA).

The analyses were performed using the SPSS 22.00 statistical software [17].

## 3. Results

### 3.1. Preliminary Analysis

EG1 and EG2 did not differ significantly in age (EG1: M = 8.18, SD = 0.45; EG2: M = 7.97, SD = 0.53; t = 1.798; *p* = 0.076), or intellectual ability (learning potential). The direct scores on a scale of intelligence (Factor G; [14]) did not significantly differ once age was controlled for (EG1: M = 19.20, SD = 5.47; EG2: M = 21.82, SD = 5.39; F = 3.453; *p* = 0.067). In contrast, the teachers of EG1 reported more problems of attention, self-control, and aggressiveness in their students than the teachers of EG2 (Table 6). The greater attentional problems in EG1 students were also seen in their execution in CARAS-R [15]. The number of net correct answers (correct minus incorrect answers), once age had been controlled for, was higher in EG2 (EG1: M = 18.93, SD = 8.43; EG2: M = 23.58, SD = 9.56; F = 4.575; *p* = 0.036).

### 3.2. Analysis of the Effect of the Intervention

**Figure 1:**
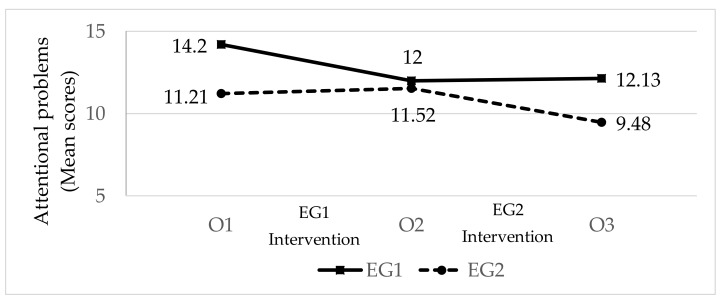
The effect of the intervention on pupils’ attentional problems (n_GE1_ = 40; n_GE2_ = 33; EG = Experimental Group; O = Observation).

*Attentional problems* (Figure 1; Table 6). At the beginning, EG1 exhibited more attentional problems than EG2. The size of the initial difference between the two groups was moderate. Following the intervention with EG1 (O2), the attention problems in this group declined moderately, while those of EG2—who had not received treatment at this time—stayed the same, thus equalizing the scores between the two groups. Following the intervention with EG2 (O3), the attentional problems in this group fell slightly, while those of EG1—who received no treatment at this time—remained level. In this manner, at the end of the study, EG1 again exhibited higher levels of attention problems than EG2, with a moderate effect size. Nonetheless, there were lower levels of attentional problems in both groups at the end of the study (O3) than at the beginning (O1).

**Figure 2:**
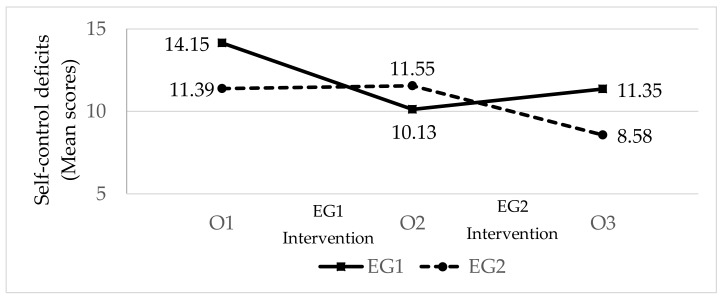
The effect of the intervention on pupils’ self-control deficits (n_GE1_ = 40; n_GE2_ = 33; EG = Experimental Group; O = Observation).

*Self-control deficits* (Figure 2; Table 6). At the beginning, EG1 presented more self-control deficits than EG2. The effect size of the difference between the two groups was moderate. Following the intervention with EG1 (O2), the level of self-control deficits in this group fell (with a large effect size), while in EG2, with no treatment, it was unchanged. Following the intervention with EG2 (O3), the self-control deficits in this group fell moderately, while the levels in EG1, with no treatment at this time point, increased slightly. Nevertheless, the levels of deficits of self-control in both groups at the end of the study (O3) were lower than at the beginning (O1).

**Figure 3:**
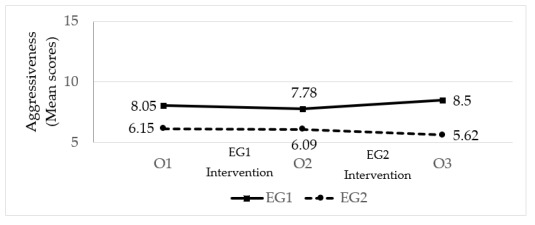
The effect of the intervention on pupil aggressiveness (n_GE1_ = 40; n_GE2_ = 33; EG = Experimental Group; O = Observation).

*Aggressiveness* (Figure 3; Table 6). At the beginning of the study (O1), EG1 exhibited more aggressiveness than EG2. The magnitude of the initial difference between the two groups was moderate. After the intervention with EG1 (O2), no significant change was seen in their level of aggressiveness, nor was there any change in EG2, who had not received treatment at this time point. At O2, therefore, EG1 continued to have a higher level of aggressiveness compared to EG2. Following the intervention with EG2 (O3), the levels of aggressiveness in this group fell slightly, while the levels in EG1, who did not receive treatment in this phase, increased slightly.

## 4. Discussion

The objective of this study was to examine the effect of a mindfulness-based intervention in a school context on attention, self-control, and aggressiveness in a sample of primary school pupils. It started with the hypothesis that the intervention program being tested (called *Mindkeys Training*) would contribute to improvements in attention and self-control, along with a reduction in aggressiveness, in pupils. The results suggest that the intervention demonstrated the expected effects, although with some caveats.

The best results were found in the *attention* variable. Following the intervention (post-test), both groups evidenced a decrease in attention problems according to teachers’ reports. In the group with a follow-up (EG1), this improvement was seen to be sustained over time. For *self-control* the results also suggest that the intervention served to reduce deficits in both of the groups studied. In fact, it was this variable in which we saw the biggest improvement after the intervention (post-test). However, this effect was less sustained over time compared to that for attention. The follow-up with EG1 showed that that self-control, rather than continuing to improve, or level off, instead worsened slightly. The effect of the intervention on *aggressiveness* was also positive, although more limited than on either attention or self-control. The data suggest that the intervention in EG2 slightly lowered the level of aggressiveness, while in EG1, it slowed its upward trend. In the follow-up, the level of aggressiveness in EG1 rose slightly. These results may be explained, at least in part, by initial differences between the two experimental groups: EG1 initially exhibited more problems of attention, self-control, and aggressiveness than EG2, as well as a greater tendency to worsen in the absence of treatment.

The results of this study are in line with previously published research on the effect of mindfulness-based training in a school context. Like the meta-analysis from Zenner, Herrnleben-Kurz, and Walach [7], in this study we found a positive, moderate-to-large effect, mainly on the cognitive variables that the intervention program targeted directly. But there was also a positive, albeit smaller, effect on aggressiveness, which the program addressed indirectly via tasks to improve attention, self-control, and awareness and the management of emotions. Previous studies have indicated the positive effect of this kind of intervention on the improvement of emotional skills [18,19], increased prosocial behavior [8], and reductions in disruptive behavior [9] and school aggression [20].

The positive results of the intervention program (Mindkeys Training) can be explained by several factors. With regard to attention, most of the tasks consist of focusing attention on a primary aim, whatever it is (e.g., breath), and staying focused on it during the activity. This practice might contribute to improved selective and sustained attention abilities. Concerning self-control, most of the tasks were designed to help students to become fully engaged in the activities they perform, even in presence of perceptual or emotional distracters. This might promote a more reflective performance. Regarding aggressiveness, the improvement in attention and self-control, which are correlates of aggressiveness [21], might lead to not only a cognitive but a socio-emotional and behavioral improvement. Most of the activities involve relaxing the body and mind, helping to focus on the present, and avoiding devoting attention to negative emotions. In addition, some activities directly aimed at improving students’ prosocial attitudes were included in the intervention program.

Furthermore, as prior research suggests, the initial training of teachers about mindfulness and involving them in the student training might positively contribute to the effect of the intervention. Firstly, the initial training sessions for teachers might have a certain positive effect on their wellbeing [22], mainly through improvements in their capacity for emotion regulation and a reduction of stress [23]. This may positively affect the way they deal with their pupils and manage classroom relationships [24,25,26], and may provide a suitable model of behavior for their pupils. Training teachers in mindfulness may therefore have a positive impact on student behavior, and may have had a positive impact on the effect of the intervention program in this study. Secondly, the pre-training activities conducted by teachers might have contributed to a better school climate. Finally, previous meta-analyses have indicated that mindfulness-based interventions in schools have tended to demonstrate more positive effects in follow-ups when trained teachers delivered the program, rather than when a program was delivered by an outside facilitator [4]. If the teacher runs the sessions that have led to improvements in their pupils, it is more likely that the improvement will continue even in the absence of an external expert.

Despite previous meta-analyses indicating that mindfulness-based interventions are usually more generally effective in adults [8] and, in the educational sphere, in adolescence rather than in middle childhood [4], our results show that this type of program (and in particular the program we tested, *Mindkeys Training*) can also be effective in children in the third year of primary school (7 to 10 years old). Nevertheless, the results from our study suggest that at these ages, a continued intervention might be especially important. One possible reason why the expected results were not found at follow-up for some variables may be that children find it more difficult to practice what they learn spontaneously or autonomously in their daily lives (in fact, the group with which we did a follow-up, EG1, exhibited greater deficits in self-control that EG2). Thus, schools encouraging the continuing use of these kinds of routines might help the positive effects of the intervention to be maintained over time.

This study makes a theoretical and practical contribution to the study of the effects of mindfulness-based interventions in a school context. From a practical perspective, we have presented in this study the structure of *Mindkeys Training,* a mindfulness-based intervention program that has demonstrated its positive effects in third year primary classrooms in improving pupils’ attention and self-control, and reducing aggressiveness. This structure may guide teachers and professionals interested in improving these variables in school, using this approach. From a theoretical perspective, the study contributes to clarifying the impact of mindfulness-based intervention programs on attention, self-control, and aggressiveness, in school contexts. In contrast to other studies which used simpler designs, the research design used in this study (*switching replications design*) allowed the control of threats to internal validity (variables separate from the treatment that could explain the observed changes) that are not always controlled, such as the effects of pupils maturing, or the repeated administration of tests [27].

This study is therefore a contribution to the study of the impact of mindfulness-based intervention in school contexts—in particular, in primary education. However, it is not without limitations. Firstly, the sample used was not very large, nor was it randomly selected. It would be useful to replicate this study in other third year primary students to check whether the same results are produced, or if not, which variables are responsible for greater or lesser effects of the program. Secondly, the results were obtained from third year primary students in Asturias (Spain). Any generalization of the conclusions to different ages or geographical contexts should be made with caution. It would be desirable to apply the intervention to other ages and contexts to check its effects. Finally, pupils’ attention, self-control, and aggressiveness were measured via a teacher rating scale. Future validations of the program should complement information from teachers with information from execution tasks, or self-reports from the students.

## 5. Conclusions

The mindfulness-based intervention program (*Mindkeys Training*) demonstrated a positive effect on the reduction of third year primary pupils’ attention problems, deficits of self-control, and aggressiveness. The effect was greater on the cognitive variables that the intervention worked on directly (attention and self-control). Attention was the variable on which the intervention exhibited the longest term effects.

## Figures and Tables

**Table 1:** The research design.

Group	Assessment Sequence				
EG1	O1 (Pretest)	Intervention	O2 (Post-test)	No intervention	O3 (Post-test)
EG2	O1 (Pretest)	No intervention	O2 (Pretest)	Intervention	O3 (Post-test)

EG = Experimental Group; O = Observation.

**Table 2:** The activities and content of the two initial training sessions for teachers.

Session	Content	Activities
1	What is mindfulness and what is if for?	Read a text on mindfulness and its usefulness [11]. Reflection and debate on what is and what is not mindfulness.
	Breathing as a resource for focusing attention.	Explanation and practice of breathing techniques.
	Conscious attention	Full awareness activity. Practical with writing and choice of a daily routine activity outside of the classroom to practice (e.g., brushing teeth).
2	Kindness and gratitude.	Read a story about the task of education: “The prince who lost his memory” [12]. Gratitude practice (related to personal and classroom life): Daily thinking about things one should be grateful for. Kindness practice: Note positive aspects of students, to use them later with the pupils. Read a story encouraging positive self-esteem in pupils “My compliments collection” [12].
	Initial pupil training	Explanation of activities to be introduced into the classroom before the start of the pupils’ 8 weeks training.

**Table 3:** The pre-training activities with pupils.

Week	Activities
1	Say hello/goodbye to each child with a look and a smile (e.g., when checking the attendance list).
2	Write a compliment for each child, praising a character trait, and leave it as a note on each desk.
3	Get the pupils to achieve 60 seconds of silence.

**Table 4:** The content of eight mindfulness-based sessions for pupils.

Session	Content	Activities
1	What is mindfulness and what use is it?	Story “Correprisas and Tumbona” [12]. Debate.
	The attentive posture	“Zipping up”.
	Attention to sound	Attention to sound practice: the Tibetan singing bowl
	Breathing	Breathing technique practice: feel the breathing in my nose.
	Attention to how I feel	“My internal weather”.
	Attention to my body	Body-scan
2	The attentive posture	“Zipping up”.
	Attention to sound	The Tibetan singing bowl
	Breathing	Feel the breathing in my chest.
	Curiosity	Story “A surprising present” [12]. Debate.
	Attention to what we see	Missing object or added object
3	The attentive posture	“Zipping up”.
	Attention to sound	The Tibetan singing bowl
	Breathing	Feel the breathing in my abdomen.
	Resilience	Story “The story of a can” [12]. Debate.
	Discover our skills and qualities	The search for my treasure
4	The attentive posture	“Zipping up”.
	Attention to sound	The Tibetan singing bowl
	Breathing	Breathing with your friend (in pairs)
	Gratitude	The gratitude and care challenge
	Caring for others	Story “Chusco, a stray dog” [12]. Debate.
5	The attentive posture	“Zipping up”.
	Attention to sound	The Tibetan singing bowl
	Breathing	Breathing with sound
	Rejection	Story “Poor winter!” [12]. Debate.
	Wisdom	Planting seeds.
	Evaluate things positively	
6	The attentive posture	“Zipping up”.
	Attention to sound	The Tibetan singing bowl
	Breathing	Breathing with stones.
	Value difference	Story “Little white bear” [12]. Debate.
	Notice details	“Favorite stone”.
7	The attentive posture	“Zipping up”.
	Attention to sound	The Tibetan singing bowl
	Breathing	Positive mantra with touch.
	Anger	Story “A walk among the stars” [12]. Debate.
	Avoid negative thinking; and promote positive language	Clouds that come and go (distancing negative thoughts and attracting desires/dreams).
8	The attentive posture	“Zipping up”.
	Attention to sound	The Tibetan singing bowl
	Breathing	Geometric breathing
	The feeling of belonging to a group	Story “Something happened in the Pleiades” [12]. Debate.
	Co-operation and kindness	Co-operative movement: a glass of water
		Co-operative mandala
		Massage in pairs

**Table 5:** The sequence of testing and intervention.

	Observation 1 (Pretest) January 2019	Intervention	Observation 2 (EG1Post-test/ EG2 Pretest) March 2019	Intervention	Observation 3 (Post-test) June 2019
EG1	Pupils: Factor G & CARAS-R Teachers: adapted SENA	Yes	Teachers: adapted SENA	No	Teachers: adapted SENA
EG2	Pupils: Factor G & CARAS-R Teachers: adapted SENA	No	Teachers: adapted SENA	Yes	Teachers: adapted SENA

EG = Experimental Group.

**Table 6:** A cross-sectional and longitudinal comparison of the levels of pupils’ attention problems, self-control deficits, and aggressiveness, as reported by their teachers (n_GE1_ = 40; n_GE2_= 33).

		O1		O2		O3	O1-O2			O2-O3		
		M (SD)	Treat	M (SD)	Treat	M (SD)	t/Z	*p*	d	t/Z	*p*	d
ATP	EG1	14.20 (4.63)	X	12.00 (5.12)	-	12.13 (5.15)	4.364 ^1^	<0.001	0.446	−0.292 ^1^	0.772	−0.024
	EG2	11.21 (4.48)	-	11.52 (5.39)	X	9.48 (4.61)	−0.513 ^1^	0.612	−0.060	4.878 ^1^	<0.001	0.385
	t	2.787		0.393		2.286						
	p	0.007		0.695		0.025						
	d	0.655		0.092		0.538						
SCD	EG1	14.15 (3.86)	X	10.13 (4.40)	-	11.35 (5.13)	7.388 ^1^	<0.001	0.965	−3.722 ^1^	0.001	−0.241
	EG2	11.39 (4.46)	-	11.55 (5.05)	X	8.58 (3.85)	−0.284 ^1^	0.778	−0.031	5.863 ^1^	<0.001	0.614
	t	2.831		-1.284		2.566						
	p	0.006		0.203		0.012						
	d	0.666		-0.302		0.603						
AGG	EG1	8.05 (4.67)	X	7.78 (3.91)	-	8.50 (4.80)	−0.560 ^2^	0.575	0.062	−2.329 ^2^	0.020	−0.144
	EG2	6.15 (2.68)	-	6.09 (2.87)	X	5.62 (2.18)	−0.073 ^2^	0.942	0.022	−2.325 ^2^	0.020	0.151
	U	464.500		482.000		414.500						
	p	0.016		0.025		0.001						
	d	0.524		0.475		0.672						

^1^ t = Student t test for related samples; ^2^ Z = Wilcoxon test. ATP = Attention problems; SCD = Self-control deficits; AGG = Aggressiveness; EG = Experimental Group; O = Observation; X = Intervention. Theoretical range of scores: minimum 5, maximum 25.
